# Cognitive decline: mechanisms and proposed role of the renin–angiotensin–aldosterone system

**Published:** 2014

**Authors:** Lionel H Opie

**Affiliations:** Hatter Institute for Cardiovascular Research in Africa, Groote Schuur Hospital and University of Cape Town Medical School, Cape Town, South Africa

## Normal cognitive functioning

Cognition is derived from the Latin ‘cognitio’, meaning the process of acquiring knowledge, with related meanings such as study, recognition, social connectivity and discovery. The most crucial components of cognition are the ability to learn and remember new information,^1^ and to function adequately in daily intellectual and interactive aspects of life.

Maintenance of normal functional cognitive activity is vitally important in everyday activities. Conversely, cognitive decline, as normally occurs during the ageing process, is a handicap. Such decline varies from moderately inconvenient benign forgetfulness to the devastating losses associated with Alzheimer’s disease and brain ischaemia.^2^

## Cognitive function as part of optimal health

Stroke, often associated with untreated hypertension, is common in Africa. In the USA, the move is towards stroke prevention, by paying attention to the blood pressure as one of life’s simple seven (LS7) health metrics (blood pressure, cholesterol, glucose, body mass index, smoking, physical activity and diet).^3^

Healthy levels of these components are in the higher ranges of the following scale: inadequate (0–4), average (5–9), or optimum (10–14) cardiovascular health. In a large USA population (22 914, 42% black) over 4.9 years of follow up, better cardiovascular health on the basis of the LS7 score was associated with lower risk of stroke. Therefore, not surprisingly, higher LS7 scores were associated with lower incidence of cognitive impairment.^4^

As expected, cognitive function may be related to blood pressure (BP) control. Starting with a population of Japanese subjects with mean age 63 years and without cognitive decline, follow up after nearly eight years showed about one in 10 had undergone cognitive decline that was associated with an increase in systolic BP (odds ratio 1.48; *p* = 0.03).5 Also of interest was the link between increases in home systolic BP and the significant association with cognitive decline (odds ratio, 1.48; *p* = 0.03).^5^

The benefit of BP reduction was endorsed by links between systolic BP ≥ 140 mmHg or diastolic BP ≥ 90 mmHg and faster decline of cognitive function over two years of follow up in 1 385 persons.^6^ Thus far there has been no formal trial on the effects of BP control on cognitive function but one large trial is planned.^7^

## Role of the renin–angiotensin–aldosterone system (Fig. 1)

**Fig. 1.**
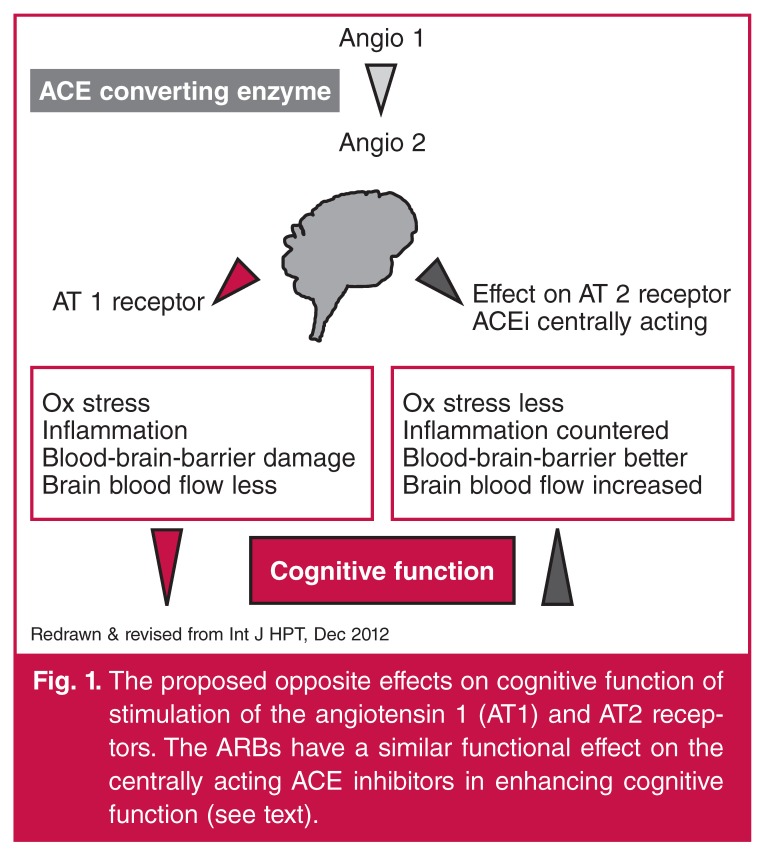
The proposed opposite effects on cognitive function of stimulation of the angiotensin 1 (AT1) and AT2 receptors. The ARBs have a similar functional effect on the centrally acting ACE inhibitors in enhancing cognitive function (see text).

The brain has an essential evolutionary role in self-protection, centered on the concept that harmful stimuli must evoke protective mechanisms. As the renin–angiotensin–aldosterone system (RAAS) has a pivotal role in cognitive impairment,^8^ logically, the use of centrally acting angiotensin converting enzyme (ACE) inhibitors should and does slow the rate of decline.^9^ These agents act on the RAAS in the hippocampus. Although statins also benefit mild cognitive impairment,^10^ they do not act centrally on the brain but on the blood–brain barrier (Fig. 1).^11^

## Inhibition of the RAAS to limit cognitive decline

Inhibition of the RAAS ranks among the best established and most used cardiovascular therapies by providing protection against hypertensive damage and heart failure, and the microvascular complications of diabetes. RAAS inhibitors have contributed to the longer life expectancy now found in higher-income populations throughout the world. However, longevity propels aging persons closer to the inevitable cognitive decline that is of variable gravity.

Perhaps unexpectedly, it is here that the RAAS inhibitors may have a special role, especially in the many elderly persons with hypertension. The two centrally active ACE inhibitors, captopril and perindopril can delay cognitive decline.^12^ Likewise, the brain-penetrating angiotensin receptor blockers (ARBs) counter cognitive decline, as in the case of telmisartan.^11^,^12^ The cellular mechanisms whereby the RAAS inhibitors act on the brain are still under study but experimentally include stress relief,^13^,^14^ and an anti-inflammatory benefit.^15^,^16^

## Conclusion

Cognitive function is one of the most important functions of the activity of the healthy mind and brain.^14^ On present data, it is reasonable to give a centrally acting ACE inhibitor or an ARB to a person with developing cognitive decline. The possible risk is that of a modest rise in blood pressure, which could be treated by lifestyle modification and/or drug therapy as needed. Solid data from large, long-term, appropriately designed trials are required.
